# Fine needle aspiration diagnosis of Rosai-Dorfman disease in an osteolytic lesion of bone

**DOI:** 10.4103/1742-6413.65058

**Published:** 2010-07-02

**Authors:** Shaoying Li, Zhijie Yan, Nirag Jhala, Darshana Jhala

**Affiliations:** Address: Department of Pathology, University of Alabama at Birmingham, Birmingham, AL, USA; # These authors contribute equally to this manuscript

**Keywords:** Bone, Rosai-Dorfman disease, FNA

## Abstract

Sinus histiocytosis with massive lymphadenopathy (SHML) or Rosai-Dorfman disease (RDD) is an uncommon but well-defined benign self-limited clinicopathological entity. It mainly involves lymph nodes. Extranodal involvement is seen in up to 43% of cases, with the most common location in the head and neck region. Primary RDD occurring in the bone is rare with only twelve cases reported in the literature to date, all diagnosed on histology except one by fine needle aspiration (FNA) cytology. We report a case of RDD diagnosed by FNA cytology in a 28 year-old female presented as an osteolytic lesion of superior pubic ramus where the differential diagnosis included a sarcoma and lymphoma. Based on the cytologic findings, a diagnosis of a RDD was considered during the rapid FNA on site with no clinical history provided. The diagnosis of RDD was further confirmed by immunohistochemical stains and histology diagnosis. Our findings show that even in the absence of a clinical history, FNA is a less invasive and a very reliable tool for the diagnosis of SHML (RDD).

## INTRODUCTION

Sinus histiocytosis with massive lymphadenopathy (SHML) or Rosai-Dorfman disease (RDD) is a rare but well-defined benign self-limited clinicopathological entity. It commonly involves lymph nodes. Extranodal involvement is seen in up to 43% of cases, the most common location is the head and neck region (75%), where the skin, orbit, nasal cavity and paranasal sinuses are the most common sites.[1,2] Primary RDD occurring in the bone is rare with only 12 cases reported in the literature to date, all diagnosed on histology except one by FNA cytology.[3] FNA is a safe, relatively non-invasive diagnostic procedure often used to diagnose neoplasm and inflammatory disease. The characteristic cytomorphological features of RDD obtained via FNA have been described in cases reports.[4–8] We report a case of RDD diagnosed by FNA cytology in a 28 year-old female presented with an osteolytic lesion of the superior pubic ramus.

## CASE REPORT

A 28-year-old woman presented with progressive hip girdle pain for several months and underwent imaging studies which revealed a destructive lytic bone lesion around the right acetabulum and proximal femur involving the superior pubic ramus. The pain was worsening with weight bearing and was controlled fairly well with pain medications. The patient had no significant constitutional symptoms. She denied any weight loss. Review of systems was unremarkable. Her past medical history was also unremarkable.

### Laboratory values

The patient’s laboratory values at the time of admission included hemoglobin of 12.2 g/dl; hematocrit of 35.3%; white blood cell count of 5,630/mm^3^; platelet count of 244,000 mm^3^ and erythrocyte sedimentation rate of 50 mm/hr.

### Imaging studies

**Figure 1:**
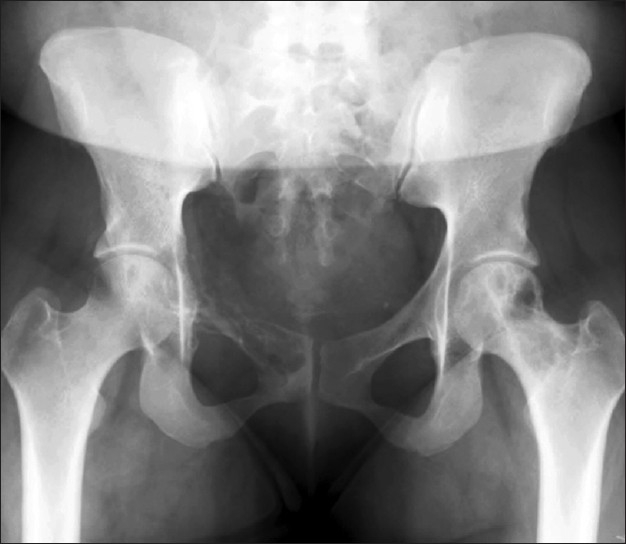
Anterioposterior pelvis radiograph of the right superior pubic ramus and acetabulum osteolytic lesion

An anterior-posterior pelvis radiograph showed an osteolytic bone lesion in the right superior pubic ramus and acetabulum [Figure 1]. A computer tomography (CT) scan revealed the same destructive lytic bone lesion around the right acetabulum and superior pubic ramus. There was marked destruction of the cortex and an associated soft tissue mass extending into the obturator internus muscle. There was no lymphadenopathy. Differential diagnosis included metastatic neoplasms versus primary bone tumors or lymphoma.

### Cytological findings

**Figure 2a-c:**
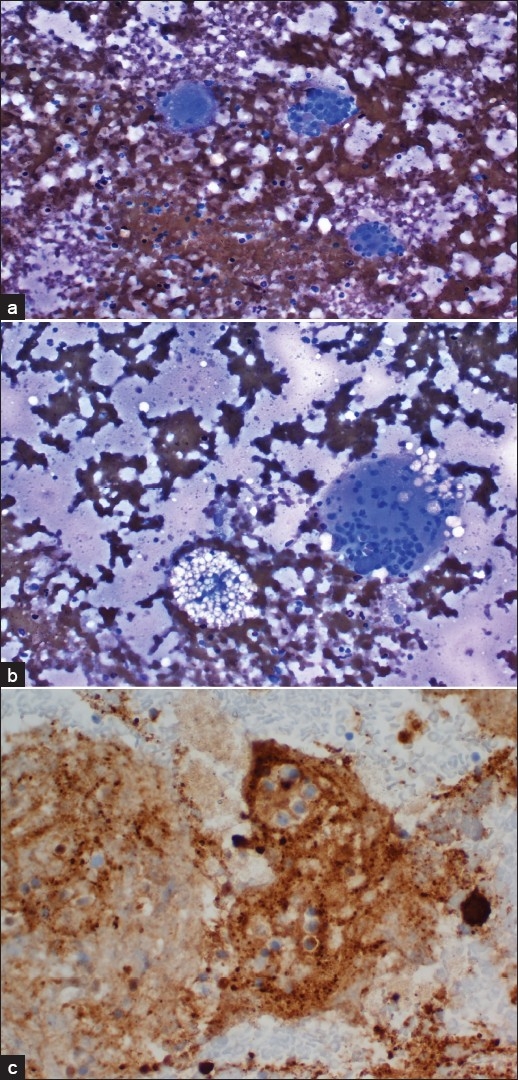
FNA of the right superior pubic ramus. a) Air-dried smear showing large histiocytes with predominant emperipolesis (contain numerous phagocytosed intact lymphocytes, neutrophils and rare plasma cells within the cytoplasm; Diff-Quik, 400X); b:)Foamy histiocytes and halos around some of the phagocytosed cells (Diff-Quik, 200×). c) Immunocytochemical stain performed on the cell block revealed strong cytoplasmic stain for S-100 protein (Immunoperoxidase stain, 400×) but negative stain for CD1a (not shown)

A CT guided FNA of the right superior pubic ramus was performed using a 22 gauge needle. Air-dried smears were stained with Diff-Quik (Baxter, Melrow Park, IL, USA). The needles from each pass along with remaining FNA samples were rinsed with Hank’s solution and used for making cell block. Rapid interpretation was given based on the finding noted on the Diff-Quik stain. The specimen was hypocellular and revealed varying numbers of large histiocytes with prominent emperipolesis. Those large histiocytes contain numerous phagocytosed intact lymphocytes, neutrophils and rare plasma cells within the cytoplasm [Figure 2a]. Fine vacuoles in the cytoplasm of the histiocytes were easily appreciated. There were polymorphous lymphoid elements and relative lack of eosinophils; with slightly increased numbers of plasma cells in the background [Figure 2b]. No pleomorphic spindle cells or monomorphic lymphoid cells were noted, excluding the possibility of sarcoma or lymphoma. Based on these findings, a diagnosis of a RDD was considered. No clinical history was provided at the time of rapid interpretation.

### Immunocytochemical studies

Immunoperoxidase stains for antibodies to S-100 protein and CD1a were performed on the cell block. The histiocytes were intensely positive for S-100 protein [Figure 2c] and negative for CD1a, supporting the diagnosis of RDD.

### Follow-up

A follow-up biopsy confirmed the diagnosis of RDD histologically. Due to progressive pain and concern for a potential future pathologic fracture, the patient was advised to undergo radiation therapy and chemotherapy that she was to pursue in a different city from where she lives. Follow- up had been lost since then.

## DISCUSSION

SHML or RDD was first described by Rosai J. and Dorfman R.F. in 1969.[9] It is a rare but well-defined clinicopathological entity. It affects all age groups, but is predominantly seen in children and adolescents and more commonly seen in males. It is a histiocytic proliferative disorder of unknown etiology characterized clinically by painless bilateral cervical lymphadenopathy in the majority of patients; accompanied by fever, leukocytosis, elevated erythrocyte sedimentation rate (ESR), and hypergammaglobulinemia.[2,10] Extranodal involvement is seen in up to 43% of cases. The most common location is the head and neck region (75%), where the skin, orbit, nasal cavity and paranasal sinuses are the most common sites.[1,2] Primary RDD occurring in the bone is rare with only 12 cases reported in the literature to date, all diagnosed on histology except one by fine needle aspiration cytology.[3] Most cases of RDD follow a waxing and waning course over years with eventually complete regression and do not require any treatment; therefore, the correct diagnosis is very important. Histologically, severely dilated lymphatic sinuses are occupied by lymphocytes, plasma cells and numerous large histiocytes resulting in partial or complete architecture effacement. The hallmark of this disease is emperipolesis or lymphocytophagocytosis, that is, many large histiocytes contain numerous phagocytosed intact lymphocytes (sometimes other cells such as plasma cells and red blood cells) within the cytoplasm. It is a non-specific but diagnostic feature of RDD. Cytological findings demonstrate a high degree of histological correlation and consistency. The significant cytomorphological features of RDD reported include cellular smears containing large histiocytes (150/200 μm) with prominent emperipolesis, halos around each of the phagocytosed cells, fine vacuoles in the cytoplasm of the histiocytes containing large amount of neutral lipid demonstrated by Oil red O stain, relatively lack of follicular center cells and eosinophils, and an increased number of plasma cells in the background.[4–8,11,12] All these features were present in our case. We agree with most other reports that the cytological features of RDD are very characteristic so that FNA cytology is a reliable diagnostic modality. However, in spite of its well-defined cytomorphological features, diagnosis of SHML (RDD) may be very challenging, especially in extranodal sites. Because of its non-specific clinical presentation and sometimes with only subtle changes or even with atypical features as well as its rarity, it may be overlooked or misinterpreted by cytopathologists. Lymphophagocytosis can be seen in many different clinical settings, of note, sinus hyperplasia, and virus associated lymphophagocytic syndromes, lymphadenitis caused by salmonella, rhinoscleroma, and histoplasmosis. Hence the differential diagnosis of SHML (RDD) in general and in particular should include the above mentioned entities. For virus associated lymphophagocytic syndromes, except different clinical manifestation such as skin rash, cytologically viral cytopathic effect is very helpful for the differential diagnosis. Rhinoscleroma is a chronic granulomatous condition of the upper respiratory tract and the presence of granulomas is a distinctive feature. Histoplasmosis will have the tiny intracellular organisms present. Additional clinical, morphological, and laboratory information on these entities will be helpful in making a correct diagnosis. The differential diagnoses also include Langerhans’ cell histiocytosis (LCH), malignant histiocytosis, and non-specific reactive histiocytosis.[13] LCH occurs mainly in children and young adults with a male predominance. It also causes lytic bone lesions. In LCH, the histiocytes have a longitudinal groove and a coffee-bean appearance. The inflammatory background consists of large numbers of eosinophils. Langerhans cells express CD1a and S-100 but lack expression of CD45. RDD cells consistently express S-100 protein and CD68, but negative for CD1a. CD1a is a more specific marker for LCH and an important feature to differentiate LCH with other histiocytic lesions like RDD. Ultrastructrally, the presence of cytoplasmic rod or tennis-racket like Birbeck granules are characteristic of Langerhans cells which are not present in histiocytes of RDD or other histiocytic lesions. Malignant histiocytosis is very rare and mainly involves extraskeletal site. The cells are large and round with abundant eosinophilic cytoplasm. Emperipolesis is not seen. The cells are positive for lysozyme, which is negative or very weak in RDD. Reactive histiocytosis is a benign entity mainly involving lymph nodes. The histiocytes in this benign entity are negative for S-100 protein. In addition, emperipolesis could be overlooked or mistaken for tingible body macrophages as seen in follicular hyperplasia with plasmocytosis and sinus histiocytosis.[14] Extracaution has to be taken when interpreting lymph node or extranodal FNA cytology smears. Rarely SHML has been identified as an isolated phenomenon in the lymph nodes affected by non-Hodgkin’s lymphoma (NHL) or Hodgkin’s disease[15] and even NHL may follow SHML.[16] In a solitary lytic bone lesion as seen in our case, giant cell tumor of the bone or osteosarcoma has to be considered in the differential diagnosis. Emperipolesis may be misinterpreted as osteoclasts or giant cells. However, other cytomorphologic features and clinical information as well as immunohistochemical stains would be beneficial in making a correct diagnosis.

## CONCLUSION

Our findings show that even in the absence of a clinical history, FNA is a less invasive and a very reliable tool for the diagnosis of SHML (RDD).

## COMPETING INTEREST STATEMENT BY ALL AUTHORS

No competing interest to declare by any of the authors.

## AUTHORSHIP STATEMENT BY ALL AUTHORS

Each author acknowledges that this final version was read and approved. All authors of this article declare that we qualify for authorship as defined by ICMJE http://www.icmje.org/#author. Each author has participated sufficiently in the work and take public responsibility for appropriate portions of the content of this article.

## ETHICS STATEMENT BY ALL AUTHORS:

As this is case report without identifiers, our institution does not require approval from Institutional Review Board (IRB) (or its equivalent).

## EDITORIAL / PEER-REVIEW STATEMENT

To ensure integrity and highest quality of CytoJournal publications, the review process of this manuscript was conducted under a double blind model (authors are blinded for reviewers and reviewers are blinded for authors) through automatic online system.
